# Prevalence and Associated Risk Factors of *Trypanosoma evansi* in Camels in Ethiopia Based on Parasitological Examinations

**DOI:** 10.1155/2020/6172560

**Published:** 2020-08-27

**Authors:** Bekalu Gerem, Muhammed Hamid, Ayalew Assefa

**Affiliations:** ^1^Abergelle District Agricultural Office, Abergele, Ethiopia; ^2^College of Veterinary Medicine, Samara University, P.O. Box 132, Samara, Ethiopia; ^3^Sekota Dryland Agricultural Research Center, Sekota, Ethiopia

## Abstract

Camel trypanosomosis (surra), caused by *Trypanosoma evansi *, is a life-threatening disease with negative impacts on health, production, and working efficiency of camels in different camel-rearing areas of the world, including Ethiopia. A cross-sectional study was carried out from November 2016 to May 2017 to determine the prevalence of camel trypanosomosis (surra) and assess the associated potential risk factors in Dubti and Asayita districts of Afar region, Ethiopia. Blood samples were collected from 200 camels. Wet film and Giemsa-stained blood smears were used for the detection of trypanosomes. Out of 200 examined, 9(4.5%) were positive for *Trypanosoma evansi*. The higher prevalence of the disease was observed in Dubti (6.97%) than in Asayita (2.63%) districts. Highest infection was observed in the age group >4 years old camels (7.47%), followed by <4 years old camels (1.07%). Previously aborted camels were found at higher risk (*P*=0.03; OR = 5.11, 95% CI = 1.174–22.317) than camels without an abortion history. There was no statistically significant difference in between body condition categories and herd size of camels with the occurrence of the disease (*X*^2^ = 3.839; *P*=0.147 and *X*^2^ = 0.718; *P*=0.698), respectively. The result of the current study revealed that camel trypanosomosis is substantially prevalent in the study area, indicating the need for designing control and prevention strategies.

## 1. Introduction

The camels (*Camelus dromedarius*) are the most numerous species of animal in the arid areas of Asia and Africa, particularly in the arid lowlands of east African counties (Sudan, Ethiopia, Somalia, Kenya, and Djibouti) [1]. Ethiopia is known for possessing the most significant number of livestock population ranking 9^th^ in the world and 1^st^ in Africa [2]. In Ethiopia, the importance of the animal is increasing from time to time both at local and global markets. Moreover, camel husbandry is the primary source of living for millions of pastoralists in the arid and semiarid zones of Ethiopia [3]. In Ethiopia, camels inhabit almost all peripheral drier lowlands that generally fall below 1,500 metres above sea level. These areas include the major parts of the Somali and Afar National Regional States and some parts of the Oromia National Regional State [4]. Ethiopia is estimated to be the third-largest camel herd in the world after Somalia and Sudan [5].

Camels are believed comparatively to be less susceptible to many of the devastating diseases that affect other livestock species, such as rinderpest, contagious pleuropneumonia, and foot and mouth disease, but yet they are affected by many other disorders [6]. Among the constraints of the disease, parasitism is one of the significant problems that affect the productivity of camels. Of these parasitic diseases, camel trypanosomosis continues to be a significant problem in sub-Saharan African countries, including Ethiopia [7].

Camel trypanosomosis, also known as Surra caused by *Trypanosoma evansi*, is the most important and serious pathogenic protozoan disease of the camel. This is transmitted mechanically by haematophagous biting flies, especially tabanids [8]. *Trypanosoma evansi* which multiplies in blood and other body fluids is the most widely spread endemic disease (surra) of camels and different domestic animals throughout the world [9, 10]. From two strains of *T. evansi*, types A and B, both are believed to be found in Ethiopia [11].

The disease is highly prevalent in Ethiopia and an important single cause of economic losses, causing morbidity of up to 30.0% and mortality of around 3.0% camels in Ethiopia [1]. In camels, the disease induces anaemia, fever, depression, dullness, weakness, and nervous symptoms and are responsible for major economic losses in terms of poor production (milk, meat, fertility, draught power, and manure) and sometimes abortion or death in case of no treatment [12]. It may occur in both acute and chronic forms; the acute form of the disease is usually fatal within a few weeks, but the chronic form lasts for years and is associated with a secondary infection [13].

Despite all its ecological and economic importance and significant role in the life of the pastoral community, until recently, the animals were neglected by researchers and development planners in Ethiopia. Moreover, there is no well-documented information about camel trypanosomiasis and associated risk factors in Afar regional state, northern Ethiopia, particularly in this study area. The current study was designed to fulfil this gap in the country and, in particular, in the study area with the objectives of determining the prevalence and associated risk factors of camel trypanosomiasis.

## 2. Materials and Methods

### 2.1. Description of the Study Area

Afar regional state is located in the Great Rift Valley, comprising semiarid rangeland in northeastern Ethiopia. According to regional estimates, the livestock population of Afar is about 10.12 million tropical livestock units, and out of this, about 859,580 (8.5%) are camels. The Afar regional state has five administrative zones, which are further subdivided into 32 districts. Pastoralism and agropastoralism are the two major livelihood ways practiced in the region. The population of the region is estimated to be about 1.2 million of which 90% are pastoralists and 10% agropastoral [14]. The study was conducted in three purposively selected districts of the central zone of the Afar region, namely, Asayita and Dubti.

### 2.2. Study Population

The study animals were indigenous one-humped camel which varies with age, body condition, and district that reared under the extensive husbandry system. Herd size was classified into three categories as small (Up to 10 camels in a herd), medium (11–20 camels), and large (>20 camels in the herd) by considering both the minimum and maximum herd size presented in the study areas [15]. The body score of camels was categorized from 5 (excellent) to 0 (very poor). Estimation of age was both on a subjective basis and based on information obtained from the owner.

### 2.3. Study Design

A cross-sectional study was conducted to determine the prevalence of camel trypanosomosis and assess the associated potential risk factors in Asayita and Dubti from November 2016 to May 2017. Owners provided information about physiological status (dry, lactating, or pregnant) and the previous abortion history of sampled animals in the herd.

### 2.4. Sampling Methods

The sampling method was supposed to be a multistage cluster sampling approach. However, due to the absence of information about variation between cluster and sampling frame, the sample size was not able to be determined using a cluster sample formula. Therefore, the representative zones, kebeles (Peasant Associations), were selected purposely based on camel population, willingness of pastoralists, and accessibility to vehicles. The primary and second stages were the sampling of zones and woredas, respectively. Selection of kebeles, herds, and individual camels within the herds was the 3^rd^, 4^th^, and 5^th^ stages, respectively. From the five administrative zones of Afar region, zone one was selected, and then two districts (Asayita and Dubti) were selected based on convenience and camel population. Accordingly, 114 (57%) and 86 **(**43%) the camel was sampled from Asayita and Dubti district, respectively. Every camel was selected from a given herd by using a simple random sampling approach.

### 2.5. Sample Size Determination

Cluster sampling is a suitable method for this study as constructing a sample frame for random sampling is not possible in the pastoral production system. However, to apply the formula, there is no information about the variation between clusters (VC) in the study areas. Therefore, it is necessary to look for other alternatives. The formula given by Thrusfield determines the size of the study animal [16] within a 95% confidence interval (CI) at 5% desired precision level:

(1) $$\begin{array}{c} n=\frac{1.962*\text{pexp}\left(1-\text{pexp}\right)}{d^{2}}, \end{array}$$

where *n* stands for the sample size, pexp is the expected prevalence, and *d*^2^ is the desired level of precision. Expected prevalence was taken as 2% as reported by Fikru et al. [17]; we calculated 30 animals to be the minimum sample size; however, to increase the precision, 200 camels were sampled.

### 2.6. Sample Collection Procedure

After physical restraining, the jugular furrow of each selected camel was cleaned with alcohol, and a jugular vein was punctured with a suitable instrument (lancet and needle). Samples were collected from 200 camels by puncturing the jugular vein into 5 ml ethylenediamene tetraacetic acid- (EDTA-) coated vacationer tubes, then kept in a cooler box, and transported to Samara University Veterinary Multipurpose Laboratory for lab activities. Thin blood smears were prepared following air-dried thin smears from the whole blood.

### 2.7. Laboratory Examination Procedures

#### 2.7.1. Hematological Examination

Blood samples were drawn into paired heparinized microhaematocrit capillary tubes up to ¾^th^ of their length. One end of the tubes was then sealed with crista seal (Hawaksly, England). The tubes were symmetrically loaded in the hematocrits centrifuge, with the sealed end outwards, and centrifuged at 12,000 rpm for 5 minutes. PCV levels of individual samples were determined on the hematocrits reader (Hawaksly, England), and the values were expressed in percentages. Animals with packed cell volume (PCV) <25% were considered to be anaemic [18].

#### 2.7.2. Parasitological Examination

For the wet film, a drop of blood was placed on a clean glass slide and covered with a coverslip, allowing the blood to spread as a thin layer of cells and then examined under the microscope to observe the motile trypanosomes. The air-dried smears were fixed in absolute methylene alcohol for 2 minutes. The slides were immersed in Giemsa stain for 20–25 minutes and washed with tap water to remove excess stain. After air drying, the slides were examined under oil immersion objective lens (40x) for the detection and identification of trypanosome species based on their morphological characteristics.

### 2.8. Data Analysis

The data were entered into the Microsoft Excel spreadsheet and analyzed with SPSS version 20. A chi-squared test and logistic regression were employed to investigate associations between infection status and risk factors. Factors identified as significant in this analysis were subsequently subjected to logistic regression analysis to examine the associations between *T. evansi* infections and potential risks.

## 3. Results

The parasite was detected in 9 (4.5%) of the camels by at least one of the three/two (wet film; thin/thick smear) parasitological methods. The difference in prevalence between the two administrative districts was not statistically significant (*P* > 0.05). However, the prevalence of trypanosome infection was slightly higher in Dubti (6.97%) than in Asayita (2.63%) (Table 1). Agewise analysis revealed that there was a statistically significant (*P*=0.029) difference among age groups. Higher prevalence (7.47%) was observed in the age group greater than 4 years old compared with that in less than 4 years age category (Table 1). The prevalence of trypanosome and body condition score was not significantly associated. However, the higher prevalence was found in poor body condition score camels (7.52%) followed by medium and good body condition score camels (2.94% and 1.36), respectively (Table 1). The prevalence of trypanosomes infection differed between herd categories and anaemia status numerically but not statistically (*P* > 0.05) (Table 2). There was a statistically significant association in the occurrence of the disease and physiological status, and abortion history of camels was (*P*=0.015) and (*P*=0.017), respectively (Table 2).

## 4. Discussion

The present study involving parasitological examinations provided strong evidence that camel trypanosomosis caused by *T. evansi* is widespread in Asayita and Dubti districts of Awsi Rasu zone, Afar Region, Ethiopia. The overall prevalence of camel trypanosomiasis in the study area was found to be 9 (4.5%). Trypanosoma evansi was the only species identified during this study, and it is reported to be the cause for camel trypanosomiasis from different parts of the world [19]. A relatively higher prevalence was reported as compared with the findings of [20–22] who reported prevalence of 3.9%, 0.3%, and 2% of *T. evansi* in camels in Jijiga Zone of Somalia, Issa (Afar), and Tigray, respectively. On the other hand, the finding of the present study is lower than that of a previous study [23, 24] which reported prevalence 72% and 17.9% of *T. evansi* in camels in Bale Zone and Jijiga Administrative Zone, respectively. This might be due to the variations in the ecology of the study areas and seasons of the year. Season has a direct effect on the distribution of biting flies, which are responsible for the mechanical transmission of *T. evansi* [25]. Since the study conducted was during the dry period and the climatic condition of the study areas were similar, the prevalence of trypanosomosis between the districts did not significantly (*P*=0.142) vary. However, the higher parasitological prevalence was recorded in Dubti district (6.97%) compared with that in Asayita (2.63%). The higher prevalence observed in Dubti district may be linked to the relative ecological variation; hence, in Dubti district, there are numerous animal watering points and big and medium-sized trees and shrubs along with a year-round river called Awash River.

The agewise comparison indicated that there was a statistically significant difference between age groups and the occurrence of the disease (*X*^2^ *=* 4.74; *P*=0.029) in which the higher infection rate was recorded above four years old camels than that of below four years old camels, 7.47% and 1.07%, respectively. This result is in line with other findings [24, 26, 27]. This might be due to heavy stress associated with their use for various purposes like transportation of goods, and suboptimal management practices may also have contributed to the higher prevalence of *T. evansi* infection noted in older camels [28, 29]. In contrast to our results, relatively higher prevalence of the disease was recorded in young age groups than adult camels [6].

Among body condition differences, a higher prevalence of *T. evansi* infection was noted in 7.52% in emaciated camels and 2.94% was in moderate and 1.3% was in good body condition camels. However, this risk factor was not statistically significant with the disease. This result agreed with that in [30].

Herd size analysis revealed that there was no statistically significant difference among the herd group and the occurrence of the disease (*X*^2^ = 0.718; *P* value = 0.698). However, relatively larger herd size was identified as a major risk factor of trypanosomosis in camels. In other studies, this risk factor was found statistically significant [30]. Accordingly, in [31], it is stated that infection rate according to herd size was of highest prevalence in herds possessing more than 20 animals more than herds possessing 11 to 20, 6 to 10, and one to 5 animals, respectively. This could be attributed to that most of large herds were located in an area with insect species known as a disease vector and more fly attacks.

A previous history of abortion analysis indicated that the infection rate was 15% in camels having the prior history of abortion and 3.3% in camels without past abortion history. Camels with a history of abortion were more than five folds at risk (OR = 5.11; 95% CI = 0.045–0.8521) to infection than camels with no history of the reproductive disorder. This result agrees with [32, 33] in which it was found that abortion could be due to the chronic form of trypanosomiasis.

There was a substantial, statistically significant difference between the prevalence of camel trypanosome and physiological status of camels. Pregnant camels were found with at high risk of infection by *T. evansi* followed by lactating and dry camels, respectively. This might be due to stress during pregnancy and lactation, which could decrease resistance in female and render them more susceptible to *T. evansi* infection [34]. On the other hand, some studies on prevalence and associated risk factor of trypanosomosis due to *T. evansi* in camel have been published but no information concerning the assessments of the prevalence of trypanosomiasis and physiological status of camels.

## 5. Conclusions

The study revealed that camel trypanosomosis caused by *Trypanosoma evansi* is prevalent in the Afar regional state particularly in the study area at relatively low levels during the study period from November to May, using parasitological techniques. The findings in this study might not reflect the real situation because the sensitivity of parasitological techniques in the diagnosis of *Trypanosoma evansi* has been reported to be low and most of the time, the disease is underdiagnosed, so the present study provides useful baseline data on the prevalence of camel trypanosomiasis in the study area. Therefore, well-organized prevention and control approaches need to be implemented. Further studies should be conducted across all seasons involving state-of-the-art techniques in detecting the organism in the study area as well as nationally.

## Figures and Tables

**Table 1:** Prevalence of camel trypanosomosis among associated risk factors.

Risk factor	Category	No. of tested camels	Positive (%)	Chi-squared	*P* value
Age	Adult	107	8 (7.47%)	4.74	0.029
	Young	93	1 (1.07%)		

Body condition	Emaciated	93	7 (7.52%)	3.839	0.147
	Moderate	34	1(2.94%)		
	Good	73	1(1.36%)		

Herd size	Small	40	1 (2.50%)	0.718	0.698
	Medium	93	4 (4.30%)		
	Large	67	4 (5.97%)		

History of abortion	Previously aborted	20	3(15%)	5.701	0.017
	Not aborted	180	6 (3.33%0		

Physiological status	Pregnant	67	7 (10.4%)	8.35	0.015
	Lactating	86	1 (1.2%)		
	Dry	47	1(2.1%)		

Anaemic status	Anaemic	98	5(5.1%)	0.162	0.687
	Not anaemic	102	4(3.9%)		
Districts	Asayita	114	3 (2.63%)	2.154	0.142
	Dubti	86	6(6.97%)	2.154	0.142

**Table 2:** Logistic regression analysis of risk factors and trypanosomosis prevalence.

Risk factors		No. examined	No. Infected	*P* value	OR	95% CI for EXP(B)
Age						
Adult		107	8	0.061	7.43	0.0912–60.59
Young		93	1			
Abortion history					
Aborted		20	3	0.03	5.118	1.174–22.317
Not aborted		180	6			
Physiological status						
Pregnant		67	7			
Lactating		86	1	0.034	0.101	0.638–45.169
Dry		47	1	0.122	0.186	0.033–8.854

## Data Availability

The data used to generate the results are available within the article.
